# The effect of midterm peer feedback on student functioning in problem-based tutorials

**DOI:** 10.1007/s10459-012-9364-1

**Published:** 2012-03-28

**Authors:** Rachelle J. A. Kamp, Diana H. J. M. Dolmans, Henk J. M. Van Berkel, Henk G. Schmidt

**Affiliations:** 1 Department of Educational Development and Research, Faculty of Health, Medicine, and Life Sciences, Maastricht University, P.O. Box 616, 6200 MD Maastricht, The Netherlands; 2 Erasmus University Rotterdam, P.O.Box 1738, 3000 DR Rotterdam, The Netherlands

**Keywords:** Problem-Based Learning, Peer feedback, Mixed-methods exploratory design, Effectiveness, Process feedback, Student functioning

## Abstract

Within Problem-Based Learning successful learning depends on the quality of cognitive, social and motivational contributions students make to the tutorial group. But at the same time, not all students in PBL automatically contribute in a high quality manner, which might impede successful group functioning. This study investigated whether peer process feedback combined with goal setting can be used to improve the quality of students’ individual contributions. A mixed-methods explanatory design, in which 74 second-year Health Sciences students participated, combined a pre- and posttest with a focus group. The results indicated that the quality of the contributions only increased for students with a below average score on the pre-test. The qualitative data confirmed that the impact of the feedback could be increased by combining individual reflection by means of goal setting with face-to-face discussion. Another suggestion is to investigate whether midterm peer process feedback is more effective for first year students, because they are still developing their tutorial behavior, as opposed to second year students.

## Introduction

Within health sciences Problem-Based Learning (PBL) is a commonly used instructional approach. The success of PBL largely depends on the quality of tutorial group functioning (Savery and Duffy 1996). Well functioning PBL tutorial groups are groups in which different viewpoints and ideas are articulated and discussed and new knowledge is constructed collaboratively (Visschers-Pleijers et al. 2006). In addition, the quality of functioning within the group is affected by the social climate within the group (Van den Bossche et al. 2006). But, because group functioning and individual contributions are largely interdependent (Lee and Roth 2007), well functioning groups can only exist by the grace of well contributing individuals within the group. In a prior study, a positive relationship was indeed found between the quality of students’ individual contributions to the problem-based tutorial group and achievement (Kamp et al. 2012). However, at the same time not all students within a PBL tutorial group automatically contribute in a high quality manner, which might impede successful group functioning. One common problem, for instance, within collaborative groups is that students tend to shirk their responsibility to other group members and rely on them to do the work (Decuyper et al. 2010). This so-called “free riding” has also been seen in PBL tutorial groups and students have indicated that they believe this has a negative effect on group functioning (Dolmans et al. 1998). Another common problem is the overly dominant student, because this might restrain other group members from giving their opinion. Furthermore, a problem in tutorial groups is that students sometimes avoid constructive conflicts (Moust et al. 2005). Students tend to merely state the undifferentiated main issues that resulted from their self-study, without explicitly discussing differences in viewpoints between students or sources. This is also called “group thinking” and is known to have a negative effect on group functioning (Decuyper et al. 2010). In order to overcome these problems, attention should be paid to the quality of individual students’ contributions in the tutorial group. The tutor plays an important role in promoting students’ learning in PBL (e.g. Dolmans et al. 2003; Dolmans and Wolfhagen 2005). He or she should stimulate students to elaborate (e.g. summarize in own words and search for explanations and contradictions) and to interact with each other. The tutor should set a positive atmosphere within the group, which will enhance collaborative learning. Dolmans and Wolfhagen (2005) investigated the interaction between tutor performance, group productivity, and learning effectiveness as perceived by students. They found that tutor performance correlated with both group productivity and learning effectiveness. In addition, formative peer feedback might be used to improve the quality of a student’s individual contributions (Dominick et al. 1997). Especially because students seem to be better able to evaluate their peers’ behaviour as opposed to their own (Eva and Regehr 2011). Therefore, it would be interesting to investigate whether and how formative peer feedback in a PBL tutorial group setting can improve a student’s contributions in the tutorial group.

According to Hattie and Timperley (2007) (peer) feedback is an important tool to promote learning. Its goal is to improve the performance of the receiver by closing the gap between the current performance of a student and a more positive and desired state (Archer 2010). With regard to its effects on performance, both Hattie and Timperley (2007) and Kluger and DeNisi (1996) conclude that in general feedback seems to have a positive effect on student performance. If students are given the opportunity to revise their performance after receiving peer feedback this seems to positively influence their domain specific performance. Adcroft (2011) points out two possible explanations for the positive effect of feedback on performance. First, feedback demonstrates what is understood by “good” behavior, and second, feedback can be used to diagnose the gap between a student’s current behavior and the desired behavior. According to Hattie and Timperley (2007) feedback can address four different levels: task-, process-, self-regulation-, and self level. Process level feedback addresses “the main processes needed to understand/perform the tasks” (p. 87) and is thought to enhance deep learning. Feedback on students’ contributions to the group processes within PBL tutorial groups can be regarded as process level feedback. Most feedback research has focused on the effect of task feedback and only little attention is paid to the effects of process feedback, especially process peer feedback to individuals within groups (Geister et al. 2006). The scarce research that has been done has demonstrated that peer process feedback has a positive effect on motivation, behavior, communication, and collaboration of group members (Dominick et al. 1997; Geister et al. 2006). It is, however, surprising that research on the effects of peer process feedback is scarce, because it is known from previous studies that students can evaluate their peers in a reliable and valid manner (Eva and Regehr 2011; Papinzcak et al. 2007a; Sluijsmans et al. 2001). Specifically within PBL tutorial groups, a previous study investigated whether students are able to rate the individual group member’s contributions in a reliable and valid way (Kamp et al. 2011). The results indicated that students were able to distinguish and rate three types of student activities within the tutorial group, namely cognitive, collaborative, and motivational activities. Cognitive activities are contributions that lead to the construction of knowledge and constructive conflicts (e.g. summarizing and correcting misconceptions). Collaborative activities contribute to the achievement of a shared learning goal (e.g. willingness to share information, being committed to the group). Motivational activities are activities from which a student appears to be motivated for group work (e.g. amount of contributions, participation).

But as promising as peer feedback seems to be, it is also evident that it is not effective by definition (Topping 1996). Instead it depends on a complex configuration of multiple variables (Topping 2010). Although solid experimental proof is often lacking (Gielen et al. 2010; Prins et al. 2006), several authors have tried to identify characteristics that affect the effectiveness of peer feedback. Gielen et al. (2010), for instance, investigated the effects of five constructiveness characteristics (relevance and specificity of the peer feedback, presence of justification, suggestion for improvement, and clear formulation) and of the accuracy of the negative comments on subsequent performance on a writing task. The results indicated that only the presence of justification (i.e. explanation of judgment) positively affected performance, but only for students with lower pretest scores. The presence of accurate negative comments also had a positive effect on performance, but this was inferior to the effect of justification. Sluijsmans et al. (2001) and Van Zundert et al. (2010) concluded that, even though students in higher education seem to be able to assess their own peers, peer assessment and feedback should be combined with training or that students should at least have experience with peer assessment. In the study by Sluijsmans et al. (2001), students were asked to rate their peers in a PBL context, students reported that they felt a need for a training prior to making judgments, especially if those judgments were negative. In general, students who have received training before they had to assess their peers seemed to be better able to assess their peers and had higher performances on subsequent tests (Sluijsmans et al. 2002). Another important aspect is to explain the purpose and goal of the feedback to the students before feedback is given and received. Hattie and Timperley (2007) call this *feed up* and argue that it is conditional to effective *feedback*. Archer (2010) agrees with this and states that, in general, effectiveness is increased when students recognize the relevance of the feedback. He also argues that it is important that the recipients agree with the received feedback. However, after the feedback is received, students need help to improve their future performance, which is called *feed forward* (Hattie and Timperley 2007). In order to improve their future performance students should be stimulated to reflect and act upon the received feedback (Gibbs and Simpson 2004). Prins et al. (2006) indicated that just providing feedback is not enough, but it should be accompanied by reflection on the feedback. Therefore the feedback should provide clues for behavioral change and focus on future functioning and goal setting to enhance the impact of feedback (Archer 2010; Hattie and Timperley 2007; Prins et al. 2006). This also implies that feedback should be facilitative instead of directive and should stimulate students’ self-monitoring during the execution of a task. A last important point is that effectiveness of peer feedback might also depend on the competence level of the receiver. In the aforementioned study by Gielen et al. (2010) for instance, the effect of certain characteristics of feedback seemed to be smaller for students with a higher than average pretest score. Therefore, it might be that peer feedback is especially effective with a low to average competence level.

One of the scarce studies on the effectiveness of peer process feedback is the study by Phielix et al. (2010), who investigated the effects of peer process feedback in a Computer Simulated Collaborative Learning (CSCL) environment. They concluded that a combination of peer process feedback and reflection increases awareness of cognitive and social behavior. This, in turn, can enhance the quality of contributions students make to the social climate within the group (Phielix et al. 2011). So, although these studies on the effects of process peer feedback combined with reflection in a CSCL environment showed promising results, studies on the effects of peer process feedback in PBL are limited. A few studies have been done that are described in the next paragraph.

First, Papinczak et al. (2007b) and Schönrock-Adema et al. (2007) investigated the attitudes students have towards peer process feedback. Students indicated that they gained insight into, and developed an enhanced awareness of the criteria of good tutorial performance after they had provided their peers with feedback. Students who evaluated their group members also indicated that they felt an increased sense of responsibility and that they believed it had improved their learning performance (Schönrock-Adema et al. 2007).

Zumbach et al. (2002) performed a study on the effectiveness of feedback on students’ individual contributions in computer supported PBL tutorial groups. They provided group members with a quantitative display of their motivation and contributions. The experimental group, compared to the control group who did not receive any feedback, showed an increased amount of contributions. In a later study Zumbach et al. (2004) performed a similar study on feedback in distributed online PBL tutorial groups. Here they combined two types of feedback: qualitative feedback on group members’ problem-solving strategies, and quantitative feedback on their contributions and their motivation. The first type of feedback appeared to result in better problem solutions, higher grades on knowledge tests, more contributions and a higher degree of reflection. The second type of feedback was especially advantageous for students’ motivation and their attitudes towards the course. Although both of Zumbach’s studies did not use peers as a source for their feedback, they support the suggestion that process feedback on students’ contributions within PBL tutorial groups has beneficiary effects.

Summarizing, it may be said that although students seem to be favorable towards peer process feedback and that (non peer) feedback on students’ contributions also seems to have beneficiary effect on their behavior within computer supported PBL groups, it remains unclear if peer feedback on students’ contributions can enhance behavior in a face-to-face tutorial PBL group.

This results in the following research questions:

1. Does midterm process peer feedback improve the quality of individual contributions to the PBL tutorial group?
2. Does this differ for students with different levels of functioning?
3. How do students perceive the effectiveness of process peer feedback and what are points of improvement as perceived by the students?

## Method

A mixed-methods explanatory design was used to address the research questions. In an explanatory design the quantitative study is dominant and is followed by the qualitative study. The qualitative study serves as an explanation and refinement of the quantitative results (Creswell and Plano Clark, 2007).

### Participants

Participants were 87 second-year students who attended a PBL bachelor program in Health Sciences at Maastricht University in The Netherlands. In this program students learn about the many factors that either promote or harm health. The intervention took place in the course ‘Learning, Cognition, and Personality’, which was required for all students. Participation in the feedback intervention was voluntary. During this eight-week course students met each other once a week for a 2-h tutorial group meeting. The students were divided over nine tutorial groups, each consisting of approximately ten students. In these tutorial groups students discuss a given problem and generate learning issues for further self-study. During the next tutorial, students discuss and synthesize their findings. All groups were guided by nine different tutors. During the first 3 weeks of the first-year of their bachelor program all students received a PBL training ‘learning and communicating in tutorial groups’. In this training, students are taught, among other things, how to give feedback to one another within the tutorial group. The next 4 weeks this remains a point of interest and reflection.

### Instruments

#### Quality of individual contributions

The quality of individual contributions was measured with the Maastricht-Peer Activity Rating Scale (M-PARS). This peer rating scale was developed and validated in an earlier study (Kamp et al. 2011) and consists of three subscales, cognitive, collaborative and motivational activities, which are believed to be conducive to successful learning in PBL. With this 14-item rating scale students can evaluate each of their peers individually by indicating how much they agree with every item on a five-point scale (1 = *completely disagree*, 3 = *neutral*, and 5 = *completely disagree*). One student has to be evaluated by at least four peers for one reliable evaluation. See Appendix 1 for the M-PARS.

#### Perceived effectiveness

Perceived effectiveness of peer feedback and its impeding and promoting factors was evaluated in two different ways. First, students were subjected to a six-item evaluation questionnaire enquiring the instructiveness and usefulness of the received feedback. These items had to be answered on a five-point scale (see Table 1 for the items). Second, a focus group was conducted with students. The goal of this focus group was to contribute to the interpretation of the quantitative results (Krueger and Casey 2009). The focus group was audio taped and led by moderator I (HB) who was provided with a topic list (see Appendix 2). Students were asked how the feedback had impacted them and several aspects, selected from Archer (2010) and related to the effectiveness of feedback, such as the structure, timing, and feedback procedure were also addressed.

**Table 1:** Mean scores and standard deviations for the items of the evaluation questionnaire filled in by the students in week 7 (1 = completely disagree, 2 = disagree, 3 = neutral, 4 = agree, and 5 = completely agree)

Items	Mean	SD
1. The feedback gave a good view of my own functioning	3.31	0.98
2. I found it instructive to receive this feedback	3.13	1.15
3. The tutor stimulated me to reflect on the received feedback	2.84	1.03
4. I formulated useful improvement goals	2.65	1.10
5. The anonymity of the feedback was warranted sufficiently	3.97	0.94
6. The tutor evaluated functioning of group half way through the course	3.56	1.18

### Procedure

Before the pretest, all students were asked to sign an informed consent form. The study consisted of a pre- (week 3) and posttest (week 7) of the quality of individual contributions within the tutorial group as perceived by group members. During this pre- and posttest students had to evaluate the contributions of their peers by completing an M-PARS on each group member. The scores were then aggregated per evaluated student. The intervention took place in week 4, when students received the peer feedback on the quality of their contributions in the form of an overview of the aggregated mean scores and standard deviations per item followed by a − − (<3.0 = insufficient), a − (3.0**–**3.5 = needs improvement) or a neutral (3.5–3.9 = average), or a + (≥4 = above average). In order to stimulate reflection on the received peer feedback, students were also provided with a list of improvement tips (Appendix 3) that they could use whenever they might score low on particular items, which was the second part of the intervention. Suggestions of appropriate improvement tips per type of activity were listed on the back of the peer feedback form (e.g. search for contradictions in the discussion and express these contradictions). The improvement tips were formulated by the researchers and were derived from the theories that were used to develop the M-PARS (see Kamp et al. 2011). It concerns theories that explain the processes that are conducive to tutorial group effectiveness (e.g. Dolmans et al. 1998; Slavin et al. 2003).

Next they were encouraged to formulate improvement goals with regard to the items that needed improvement. Tutors were instructed to stimulate students to reflect on the feedback and to discuss group functioning in general after the feedback was received. But since students were instructed that the data would be analyzed anonymously and would not be reported to their tutors, individual feedback results were not available for the tutors and also not discussed. Because there were no more tutorial group meetings after the data of the posttest were analyzed, students were told that they could receive their feedback on the posttest by e-mail on request. When the course was completed, the focus group was conducted to explore students’ perceptions of the effectiveness of the peer feedback. Participants were gathered by means of purposive sampling; one student per tutorial group was invited to participate in the focus group by e-mail.

### Analyses

Students were first divided into three groups based on their initial score on the M-PARS (low score: <33 %, average score: 33–67 %, and high score: >67 %). In order to assess the impact of the intervention on the three groups a mixed between-within subjects analysis of variance was conducted with total M-PARS score as dependent variable.

For the quantitative data of the evaluation questionnaire means and standard deviations were calculated. The audio taped data from the focus group was summarized by moderator II (RK). This summary was send to all students who participated in the focus group. They were asked to determine the accuracy and, if necessary, to give corrections.

## Results

### Quality of contributions

Of the 87 students, 74 students received peer feedback (response 85 %). Within one tutorial group only three students signed the informed consent form. This group (nine students) had to be removed from the study because a minimum of four evaluators per student is required for a reliable evaluation. The other four students that dropped out of the study attended too few tutorial meeting, making it impossible for their group members to evaluate them in a reliable and valid way.

**Fig. 1:**
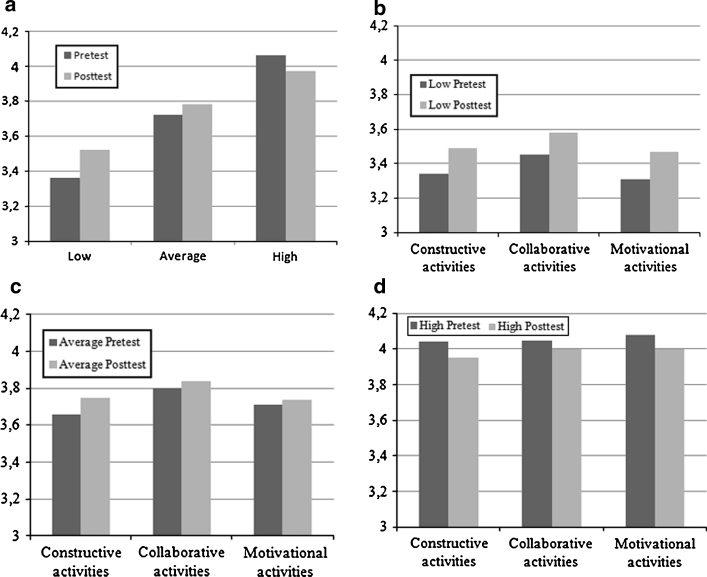
Pre- and posttest mean scores on the three subscales of the M-PARS for **a** the three groups, **b** the group with a low initial score, **c** the group with an average initial score, **d** the group with a high initial score

A mixed between-within subjects analysis of variance was conducted to evaluate the impact of the feedback intervention on the total score on the M-PARS for the three different groups (low score: N = 24, moderate score: N = 25 or high score: N = 25 on the M-PARS pretest). There was a significant interaction effect between group and time, Wilks’ Lambda = 0.88, *F* (2, 71) = 4.70, *p* = 0.012, partial eta squared = 0.12. The main effect comparing the three groups was also significant, *F* (2, 71) = 60.93, *p* < 0.0005, partial eta squared = 0.63. There was, however, no main effect for time, Wilks’ Lambda = 0.98, *F* (1, 71) = 1.66, *p* = 0.20, partial eta squared = 0.02. These results indicated that there was a difference between the three groups with regard to the effectiveness of the intervention. Therefore, three separate paired samples t-tests (one for each group) were conducted. Only the group with an initial low score on the M-PARS showed a significant, but small increase in their quality of individual contributions (M = 3.36, SD = 0.15; M = 3.52, SD = 0.33), *t*(23) = −2.29, *p* = 0.031). The results of the pre- and posttests of the three groups are displayed in Fig. 1a. Figure 1b, c, and d show the results per subscale (constructive, collaborative, and motivational) for each group (low, average, and high score).

### Evaluation questionnaire

Of the 74 students who received feedback in this study, 68 students filled in the evaluation questionnaire (response 92 %). Means and SDs were calculated for all items of this questionnaire (see Table 1). The following norm was kept: a mean below 3.0 was considered as insufficient, a mean between 3.0 and 3.5 implies that there is room for improvement with regard to the concerning item, and a mean of 4.0 or higher is considered as good. As can be seen in Table 1, the scores on the evaluation items differed between 2.65 and 3.97. The following 2 items scored below 3. Firstly, students reported that they did not formulate useful improvement goals (M = 2.65, SD = 1.10). In addition, students perceived that the tutors did not sufficiently stimulate them to reflect on the received feedback (M = 2.84, SD = 1.03). Students also indicated that the feedback gave them a reasonably good view of their functioning, but this could still be improved (M = 3.31, SD = 0.98). Some students also felt that the instructiveness of the feedback could be improved (M = 3.13, SD = 1.15). These result, therefore, suggest that there is room for improvement.

### Focus group

One student per tutorial group was invited to participate in the focus group and seven students agreed to participate. All seven students signed an informed consent form. A short summary of the results of the focus group can be found below.

Students indicated that giving feedback made them more aware of what was considered ‘appropriate tutorial behavior’ and how individual contributions influence the functioning of the group. The distinction between the three types of tutorial contributions (cognitive, collaborative, and motivational) contributed to this awareness. It also made them more critical to each other.

I think that, by looking at the quality (of contributions), it made everybody more aware of the quality level that is asked from students, especially because of the different aspects (of good tutorial behavior) that were identified.

I felt it had a positive effect on the sense of belonging to the group.

Students indicated that there was a strong relation between the functioning of the group in general and their willingness to improve their own performance in the tutorial group. Students in ‘good functioning’ groups felt that receiving the feedback had had a positive influence on their own performance as well as the functioning of the group as a whole. One student even said that it had improved her sense of belonging to the group. Students within ‘poorly functioning’ groups were less motivated to improve.

I think that it (quality of the group) also influences your own performance in the tutorial group…, because if nobody says anything and you have already read out everything for the last three tutorial meetings, by the fourth time you will think: Figure it out for yourself.

Students felt that the time between receiving the feedback and the end of the course was a bit too short to improve their contributions to the tutorial group. They felt that the effectiveness of the peer feedback would have improved when the course would have been longer.

In general students felt that the numerical design of the feedback (item mean followed by a − −, − or +) was very convenient, and they found it interesting to learn from the written feedback and improvement tips how they could improve whenever they received a below average score on an item. Therefore students felt that the improvement suggestions were useful. With regard to the formulation of improvement goals, students reported that this was not always taken very seriously, especially since the formulated improvement goals did not have to be handed in. Not all students recognized the use of formulating these goals. Other students reported that they had kept their goals in mind and were curious whether they had improved on the posttest.

I would have been very interested in the second feedback score, to see whether or not I improved.

The participating students were asked if they had any points for improvement. Four suggestions were proposed by the students.

First, students felt that the impact of the feedback could have been greater if the advantage of receiving this feedback would have been more emphasized beforehand. They indicated that they would be more motivated if the relation between the quality of students’ contributions and achievement had been more stressed.

The ‘what’s in it for me’ was a bit missing. It would have been better if it was emphasized that this feedback focuses on your functioning or your points of improvement.

Second, the participants thought the peer feedback would be more effective earlier on in the curriculum. According to them, first-year students lack a clear picture of their own individual contributions within the tutorial group and peer feedback could contribute to the illustration of their own functioning. According to several students this could stimulate students to participate more seriously in PBL tutorial groups. Second-year students are already more or less set in their ways.

Now (*in the second year*) you have already found your ways in the tutorial group. First-year students still have to develop their ways and if you then already hear that you do not contribute enough, and that this and this is good, but this and this can improve……I also think that first-year students take the feedback much more serious, because they still apply the seven-jump.

Third, as was already stated, students indicated that they sometimes wondered why they were rated below the mean on a certain item and they expressed the desire to receive some verbal clarification from their peers on their feedback. They believed that the feedback would have had more impact with this verbal clarification.

If you personalize it (*the feedback*) you could explain and nuance it. If you receive it only on paper you tend to shove it aside, but if someone tells you, you want to do something about it.

Some students, however, appreciated the anonymity of the peer feedback. They felt that publicly discussing the feedback would have a counterproductive effect because students would be giving more socially acceptable ratings. These students indicated that they had rated their peers in a critical, but honest way because they knew they would remain anonymous.

I think it’s counterproductive (to openly discuss the feedback). If you fill in the feedback rating scale anonymously people dare to be more honest.

I have filled it in very critically, but if it hadn’t been anonymous I might not have filled it in at all.

Fourth, even though students felt the improvement suggestions were useful, they thought that the improvement tips could have been more attuned to their personal score. Now everybody received the same tips and had to pick out the ones that were applicable to them.

## Discussion

The purpose of this study was to investigate whether or not peer process feedback combined with goal setting could improve the quality of students’ individual contributions within the tutorial group. In order to answer these questions a mixed-methods explanatory design was used, in which the design of the quantitative study was dominant and followed by the qualitative study. The qualitative study served as an explanation and refinement of the quantitative results (Creswell and Plano Clark 2007). Based on the quantitative data it can be concluded that, with the exception of the students with a low score on the pretest, this peer feedback intervention does not seem to improve the quality of students’ individual contributions in the tutorial group. So it seems that only the students who contribute in a poor way seem to benefit from this peer process feedback. The fact that only low performing students seem to improve their individual contributions is in line with the findings of Gielen et al. (2010). They also found that peer feedback is more effective for students with a low to average competence level as opposed to students with a high competence level. The data from the evaluation questionnaire confirmed that the instructiveness of the feedback could be improved. The focus group clarified why the feedback was not effective for the majority of the students, even though students felt that the peer feedback had made them more aware of what good tutorial behavior consists of. This resulted in a number of suggestions for improving the effectiveness of the intervention. First, there was a need for a face-to-face clarification and discussion of the received feedback. In the focus group students indicated that by discussing the feedback as a group they would be more motivated to process the feedback. Archer (2010) agrees that feedback is best discussed face-to-face, but for privacy reasons this was not done in this study. Second, more emphasis should be put on the stimulation of goal setting and reflection. In the evaluation questionnaire students already indicated that they did not formulate useful improvement goals. The reason for this was that students felt a need for more personalized improvement tips instead of the general tips and that the formulated goals were not monitored in the tutorial group. Thus, students should be stimulated more to set goals for, reflect on and monitor their contributions throughout the tutorial group meetings, especially since goal setting and reflection are important conditions for (peer) feedback to be effective (Archer 2010; Gielen et al. 2010; Prins et al. 2006). Third, more attention should have been paid to the feed up (explain purpose and goal of the feedback), which is also conditional to effective feedback (Hattie and Timperley 2007). Students indicated that, if the purpose and goal had been more emphasized, they believed the feedback would have been more effective. Fourth and last, peer feedback was thought to be more effective for first-year students, since they still have to develop their tutorial behavior. Second-year students believed they were already more or less set in their own ways, which made them less susceptible to the feedback.

Based on these findings, one could conclude that the treatment in this intervention needs to be strengthened in order to produce a behavioral change within students. Besides the aspects that were identified by the students themselves, another explanation might be that students simply did not have enough tutorials left after receiving the feedback in order to work on the improvement of their contributions. A last explanation could be that students tend to only focus on the items they received a below average score on and focused less on the aspects that scores relatively high.

Although this study provided rich insights in why and when peer process feedback might be effective in terms of improving students’ individual contributions in tutorial groups, this study does also has some important limitations. One of these limitations is that the quantitative results could also be explained by regression towards the mean because both the low and high score group trended towards the mean. The low score group did, however, show a significant and greater gain score than the high score group. In order to entirely refute this explanation and to draw solid conclusions about the effectiveness of this intervention and the effect of the different aspects discussed in the focus group, in future research experimental and control groups should be compared (Van Zundert et al. 2010). In such a study the students in the control group should not receive feedback or feedback in a more stripped form. Nevertheless, the aim of this study was not only to explain *if* peer process feedback is effective but also to clarify *why* and *when* peer process feedback might be effective. Another aspect that is worth investigating in an experimental study is the effect of actively involving the tutor in the feedback process. This is interesting because, as was mentioned in the introduction, there is an interaction between tutor performance, group productivity, and learning effectiveness. Another important limitation is that the conclusions of this study are based on students’ perceptions and not on observed behavior. Although peer evaluations are seen as valid and reliable information (Eva and Regehr 2011), it would be interesting to explore whether peer feedback also affects achievement scores.

## Appendix 2: Topic list used for the focus group

The central question presented to the students was: *How do you perceive the effectiveness of the peer feedback and what are the impeding and promoting factors?* This question was divided into four sub questions:

1. What do you think of the feedback? (timing, structure, anonymity, improvement points)
2. How did the feedback influence your individual and group functioning?
3. What were promoting and impeding factors for the effectiveness of the feedback?
4. Do you have suggestions for improvement?

## Appendix 3: Tips for improvement

**Table:**

	Definition	Improvement tips	Goals for personal improvement (min. 1, max. 3)
Cognitive contributions	Contributions that contribute to the Construction of new knowledge and the recognition of misconceptions in one’s own knowledge	Summarize the answer to a learning goal in your own words during the discussion. Search for contradictions within the discussion and express these. Explain the subject matter with an example. Use an example from daily life and not from the literature. Identify differences and similarities between different concepts. Report your findings during the discussion without checking your notes. Indicate what is unclear to you or what you are in doubt of.	
Collaborative contributions	Contributions that contribute to a good social climate within the group	Make sure your answers or information is in keeping with the previous comments or question. Look someone in the eye when you are talking to them. Repeat long answers of group members shortly in your own words. Make sure you are well prepared when come to the tutorial group meeting.	
Motivational contributions	Contributions that show a student is motivated to participate	Be the first one to start the discussion by reporting your findings. Be the first one to start the brainstorm by telling what you already know. Adopt an active attitude during the tutorial group discussion (sit up straight, hands on the table, open posture). Talk with a clear voice, watch your intonation.	
